# The Growth and Scope of Voluntary Hospitals in London

**Published:** 1897-10-16

**Authors:** 


### THE GROWTH AND SCOPE OF VOLUNTARY HOSPITALS IN LONDON.

(From a Correspondent.)

XIII.—SPECIAL HOSPITALS—(*continued*).

Two distinct lines of specialism early became apparent in the institutions to which this description may be applied. There were, in the first place, institutions intended for a special class of patients, lying-in, fever, or cancer cases, for whom accommodation could not conveniently be provided in general hospitals. In this form of specialism the doctors were tied down to no one particular form of treatment; they regarded the association of so many sufferers from one and the same disease as an opportunity for study likely to modify their methods, and were always on the watch to increase their knowledge and sharpen the weapons wherewith to combat the special malady, and its baffling intricacies of symptom.

The second form of specialism is one peculiarly attractive to the general public, and London has never been without some object-lesson in its development. It aims at cure by some special form of treatment, by preference elemental, as earth, air, fire, or water, but meat or tar, or even faith, may serve as the peg from which to hang a code of the minutest laws, and in every case the popular impulse is to establish an institution in which to watch results.

Such an enterprise was the London Electrical Dispensary established in 1793 "to afford a new benefit to the lower orders of mankind." Far more imperfectly understood than now, the phenomena of electricity presented themselves in the dawn of investigation as little short of miraculous to the lay mind, and credulity laid eager hold on the hopes of a panacea offered by eager students of its mysteries. That it relieved and cured "many, very many diseases" was doubted by few, and so cheap was the process that an average of 350 patients were annually treated at a cost of £150, or less than 10s. a head. This class of institution is seldom long-lived, and would call for no remark in a record of voluntary hospital work were it not that similar empirical charities are started every few years in London, attract public attention, flourish beyond expectation for a period, and fall slowly into disrepute and decay. It is customary to pounce on them as an evil sign of the times, and the voluntary hospital system is sometimes invoked as responsible. Whereas the art of quackery is at least as old as the science of medicine, and the charitable have in all ages delighted to comfort the stomachs of the afflicted with the one special nostrum to which they have pinned their belief.

Not all institutions for cure by special methods are open to the charge of empiricism.

A valuable cluster of dispensaries rose into being with the beginning of this century for the sole purpose of supplying and administering the newly-discovered vaccine lymph to all who would submit to the process. First introduced into London practice under Dr. Woodville at the Small-pox and Inoculation Hospital, January, 1799, the new method of inoculating for cow-pock (as it was first called), in lieu of the terribly dangerous inoculation with small-pox, sprang into immediate favour, and by December in the same year the Vaccine-Pock Institution was founded to dispense vaccination and vaccine matter. It is true that the confident declaration of the first promulgators, "that those who have undergone the cow-pox are for life unsusceptible of the small-pox," was early discredited by statistics, and a controversy, still unextinguished, and not excelled in bitterness throughout the whole course of medical history in this country, was gradually roused over the whole question. But the zeal of its promoters was only fanned by opposition. In 1803 the "Royal Jennerian Society for the Extermination of Small-pox" was established under the highest patronage, and a central institution was founded in Salisbury Square for vaccine practice. Stations were established in twelve separate districts in London for vaccination of the poor, and the society continued its work until, on its final dissolution in 1808, the Government assumed the direction of the several stations for vaccination, and established the National Vaccine Establishment, conducted by the principal officers of the Royal Colleges of Physicians and Surgeons. Long before this nearly all the dispensaries in London had added a vaccination branch to their usual lines of practice, and it is abundantly clear that early in the century no Londoner need have remained unvaccinated for want of proper facilities for the process. These vaccine charities, fleeting in their existence, though not in their effects, are again a type of what has been seen since in the founding of institutions dealing with experimental medicine. Such efforts of enthusiasm, however coolly they may be eyed at the time by those who must bear the burden of supporting the less captivating routine work of the general hospital, can be productive at worst of little harm; unsuccessful they perish unnoticed, while if the right clue be possessed they must in process of modification become absorbed in the general sum of medical knowledge and practice.

A form of special treatment which led to quite unsuspected developments was the sea bathing devised by the indefatigable Dr. Lettsom, to whom the medical charities of London owe so large a share of their wisdom and resource. Always convinced of the excellent effects to be wrought by fresh air and cold water, he took but another step in advocating the tenfold additional benefits to be got from sea breezes and sea bathing. Under his immediate direction a house was built for the reception of patients at West Brook, "near Margate," and the Sea Bathing Infirmary, which was to be a pioneer of health to millions of sufferers since, was established in 1796. "Essential for the relief of the diseased poor in inland counties, and particularly for the poor of London and its environs," Dr. Lettsom deemed it also to be "a proper supplement to the hospitals, a necessary link in the system of charities for the benefit of the indigent inhabitants of the metropolis." The difficulty of transport—the patients were for many years conveyed to Margate by sea—prevented any rapid extension of this form of charity, and it will be found that convalescent homes did not attain to Lettsom's ideal of the "necessary link" until the railway and cheap fares abolished the grave drawbacks which in the early part of this century rendered long journeys an impossibility to the sick and poor. First to follow suit were the authorities of St. George's Hospital, who, in 1809, instituted a new branch of work under the title of the Charity for Convalescents of St. George's Hospital. Much of the scope of this convalescent society was identical with the work of the Samaritan Society of the London Hospital, and consisted in the purchase of "flannel waistcoats," linen, and instruments for out-going patients. But its more important province was the payment of expenses of removing into the country, of boarding convalescents out until able to work, and of sending them to the hospitals at Bath or Margate, so that this institution may well claim the title of immediate successor to the Margate Infirmary for Convalescents. It was after all the Samaritan Fund at the London Hospital which acted as pioneer in the now indispensable work of taking thought for the out-going patient. Again we find Dr. Lettsom foremost in recommending that such societies should be generally adopted. He pointed out that Samaritan Funds were no novelty, but a mere revival of the humane provisions of the Royal Hospitals, under which one of the main objects of Bridewell was declared to be that of providing a shelter "for the sore and the sick when they be cured," that they may be protected and employed until their entire recovery. The London Hospital Samaritan Society, founded in 1791, was liberally supported, and though convalescent aid did not at this time form part of its plan the poor patients were supplied with necessary clothing, tools, or surgical appliances, their journeys home were paid, and money for their support until full recovery was ensured was frequently provided. The immediate cause of the foundation of this fund was a little incident observed by one of the early governors of the charity. A respectable poor man was discovered by this gentleman lying helpless by the roadside near London with a pair of crutches by him, on which he had started to walk back to his home in Gloucestershire after having been discharged as cured from the London Hospital. The spectacle of his sufferings, without money and without strength to continue his journey, made a deep impression not only on the spectator but on all to whom he related the story of his protégé, and effectual means were taken to prevent the recurrence of similar hardships.

One more great branch of medical charity owes its initiative to this period. The great societies for affording surgical appliances to the poor were not founded till many years after, nor was there at this time much apparatus invented of a character likely to be serviceable to working people. Crutches, artificial limbs, and the like, were cumbersome in the extreme, and the more delicate appliances which now relieve countless sufferers were unknown. Two societies, however, were in existence for providing trusses. The first Rupture Society, instituted in 1796, was intended, like so many charities of the day, specially for the benefit of discharged soldiers and sailors, although other persons were not excluded from its benefits. This society did not last long; but on its dissolution the New Rupture Society was founded, with similar aims, under new management in 1805, and the work done was further supplemented by the City Truss Society for the Relief of the Ruptured Poor, which was instituted two years later. Although medical treatment formed a part of the plan of these charities, yet as the principal part of the undertaking consisted in providing the patients with the apparatus necessary to relieve their sufferings, it may fairly be claimed that we have in these the first surgical aid society of the metropolis.

Thus every form of special charity had been thought out, and was in working order by the beginning of this century. The work of inauguration was well nigh complete. It remained to organise, to develop, to bring to perfection.

